# Editorial: Methods in structural biology: Cryo-electron microscopy

**DOI:** 10.3389/fmolb.2022.1041386

**Published:** 2022-11-10

**Authors:** Zongli Li

**Affiliations:** Harvard Cryo-EM Center for Structural Biology, Blavatnik Institute, Harvard Medical School, Boston, MA, United States; Department of Biological Chemistry and Molecular Pharmacology, Blavatnik Institute, Harvard Medical School, Boston, MA, United States; Howard Hughes Medical Institute, Harvard Medical School, Boston, MA, United States

**Keywords:** editorial, cryo-electron microscopy, Cryo-ET, methods in structural biology, sample preparation

Cryo-electron microscopy (cryo-EM) has evolved into an important method for determining the high-resolution structure of proteins and protein complexes (Kühlbrandt, 2014; Cheng et al., 2015). It is also used for *in situ* studies of lower-resolution cellular superstructures (Wan and Briggs, 2016). Further to these applications, increasing numbers of researchers are starting to use cryo-EM to address their biological questions, and are also contributing to the field in terms of method development and structure determination. This Research Topic on “*Methods in structural biology: Cryo-electron microscopy*” in the journal Frontiers in Molecular Biosciences aims to reflect the most recent developments and advances in cryo-EM sample preparation, data collection, image processing, and practices for running a shared cryo-EM facility.

Radiation damage is one factor that limits the resolution of three-dimensional structures of biological specimens when using cryo-EM. In their contribution to the collection, Shi and Huang thoroughly compare radiation damage assessments using single particle analysis (SPA) and micro-crystal electron diffraction (MicroED) (Dan and Rick). The minimum electron dose for reducing the high-resolution limit determined by SPA was tenfold higher than that measured by MicroED. The authors also propose strategies for collecting high-resolution data using SPA and MicroED.

Cryo-EM specimen preparation is the current bottleneck that impedes the broader use of cryo-EM, and many groups have been working hard to improve this. Common issues with the vitrification process include poor/non-uniform distribution of protein molecules (particles), preferred orientation, protein denaturation/degradation at the water-air interface, and high background noise from thick ice. One of the techniques developed to improve cryo-EM specimen preparation is the use of support films made of graphene and its derivatives. In their review, Fan and Sun discuss the advantages of graphene grids over conventional holey carbon film grids, the functionalization of graphene support films, how to make graphene grids, and the origins of pristine graphene contamination (Hongcheng and Fei).

Conformational heterogeneity of a biological molecule is a prerequisite for it to be able to perform its functions. In cryo-EM, specimen heterogeneity not only presents the challenge of obtaining high-resolution structure but also provides the opportunity to determine multiple structures of the same molecule/complex, with different conformations, from the same dataset, allowing investigation of the conformational changes associated with its biological functions. Hybrid electron microscopy normal mode analysis (HEMNMA) was first developed in 2014 (Jin, et al., 2014) to analyze continuous and large-scale conformational changes in biological specimens studied using cryo-EM. The technique determines the conformation, orientation, and position of the complex in each single particle image, combining the image analyses used in SPA and normal mode analysis (NMA) (the directions of motion simulated for a given atomic structure or EM map), which in turn allows the determination of the full conformational space of the complex but at high computational cost. The study by Ilyes Hamitouche and Slavica Jonic offers an improved version of HEMNMA (Ilyes and Slavica), referred to as DeepHEMNMA, that speeds up the original method by combining it with a residual neural network (ResNet)-based deep-learning approach. The authors demonstrate the performance of DeepHEMNMA using synthetic and experimental data. Two-dimensional classification has played an important role in getting rid of junk particles from large datasets, and in sorting out the conformational heterogeneity of proteins/protein complexes in a dataset. However, the process can take a long time to complete if only central processing units (CPU) are used, especially for large datasets or large box sizes, which are common in today’s cryo-EM environment. Fabian et al. presented a graphics processing unit (GPU)-accelerated version of iterative stable alignment and clustering (ISAC) (Yang, et al., 2012) that enables users to produce high-quality two-dimensional class averages from large datasets on a single desktop computer equipped with affordable consumer-grade GPUs, such as Nvidia GeForce GTX 1080 TI cards. With only two such cards, GPU ISAC can match the performance of twelve high-end cluster nodes (Fabian et al.).

Cryo-electron tomography (cryo-ET) has drawn much attention in recent years, and people believe it has great potential in the fields of cellular and structural biology. In this regard, Paula Navarro has provided an overview of current hardware and software developments that allow quantitative cryo-ET studies, and discussed the limitations of cryo-ET and how to overcome them to unleash its full power (Paula).

Despite the wide applications of cryo-EM, which are due, especially, to its potential to revolutionize structural biology and new drug development, the high monetary and human resource costs involved with establishing and maintaining a high-end cryo-EM facility limit its accessibility. As a result, governments, universities, research institutes, and pharmaceutical companies around the globe have established high-end cryo-EM centers that provide access to researchers for free or at a reduced cost (Zimanyi et al., 2022). How these shared cryo-EM facilities can be run efficiently and accessed by diverse user groups presents many challenges, especially for centers that do not have well-trained and experienced staff. In their contribution to this Research Topic, Walsh et al. present a practical routine for running a research-oriented shared cryo-EM facility, developed by the Harvard Cryo-EM Center for Structural Biology. From user training in sample preparation to data collection to help facilitate biology-focused research projects, the authors share their experiences and practices, providing valuable resources for other cryo-EM facilities (Richard et al.).
